# Changes in fatty acid and mineral profiles of the turkey egg yolk-yolk sac complex during embryonic development

**DOI:** 10.1016/j.psj.2026.107593

**Published:** 2026-08-08

**Authors:** Leila Fathi, Maghsoud Besharati, Zabihollah Nemati, Reza Asadpour, Lucrezia Forte, Aristide Maggiolino, Gerardo Centoducati, Giusy Rusco

**Affiliations:** a Department of Animal Science, Ahar Faculty of Agriculture and Natural Resources, University of Tabriz, Tabriz 5166616471, Iran; b Department of Animal Science, Faculty of Agriculture and Natural Resources, University of Mohaghegh Ardabili, Ardabil 5619911367, Iran; c Department of Clinical Science, Faculty of Veterinary Medicine, University of Tabriz, Tabriz 5166616471, Iran; d Department of Veterinary Medicine, University of Bari Aldo Moro, 70010 Valenzano BA, Italy

**Keywords:** Turkey embryo, Yolk sac, Egg incubation, Fatty acid, Mineral

## Abstract

The yolk–yolk sac complex is the main nutrient source for the avian embryo and undergoes marked compositional changes during incubation. However, information on mineral and fatty acid dynamics in turkey eggs remains limited. This study evaluated changes in mineral concentrations, absolute mineral contents, and fatty acid composition of the turkey egg yolk–yolk sac complex during embryonic development. A total of 200 fertile eggs from 56-week-old native turkey breeders were incubated under standard conditions. Samples were collected at days 0, 8.5, 18.5, 23.5, and 28 of incubation. For each time point, six pooled replicates were prepared, each obtained from five eggs. Mineral concentrations and absolute contents were determined by ICP-OES, whereas fatty acid methyl esters were analysed by GC-FID and expressed as percentages of total identified fatty acids. Incubation time significantly affected the concentrations of Ca, Cu, Fe, K, Na, P, Sr, and Zn, while Mg and Mn concentrations were not affected. Calcium and Sr concentrations increased toward hatch, whereas P concentration decreased markedly. Absolute mineral contents showed distinct patterns: Ca increased after an initial decrease at day 8.5, Sr peaked at day 23.5, and P, Fe, K, Na, Cu, and Zn generally declined by hatch. The relative fatty acid profile was also affected by incubation time. Palmitic and oleic acids were the predominant fatty acids throughout incubation. At hatch, myristic, palmitic, and linoleic acids represented greater proportions of the residual fatty acid pool, whereas the relative proportions of oleic and arachidonic acids were lower. These results indicate that the turkey yolk–yolk sac complex is dynamically remodeled during embryogenesis, reflecting mineral mobilization and changes in the relative availability of fatty acids before hatch.

## Introduction

During incubation, the avian embryo depends almost entirely on nutrients already deposited in the egg. These nutrients must support growth, tissue differentiation and the final preparation for hatch. The yolk therefore should not be regarded only as a passive nutrient store. Together with the yolk sac membrane, it participates in nutrient digestion, absorption, metabolism and transfer to the embryo. The yolk sac membrane expresses genes related to digestive enzymes and nutrient transporters, confirming its active role in the mobilization of yolk-derived nutrients during embryogenesis (Yadgary et al., 2011). Lipids are the main energy source for the avian embryo and also provide structural components for developing tissues. Yolk fatty acids are mainly incorporated into triacylglycerols and phospholipids, which are progressively mobilized during incubation and used for energy production, membrane formation and the synthesis of bioactive molecules (Speake et al., 1998; De Carvalho and Caramujo, 2018). In turkey embryos, most yolk lipid transfer occurs during the final phase of incubation, when embryo growth and energy demand are particularly high (Ding et al., 1995; Ding and Lilburn, 1996). For this reason, following changes in yolk fatty acids during incubation can help describe when lipid fractions are mobilized, remodelled or retained in the residual yolk at hatch. Minerals also have specific roles during embryonic development. Calcium and phosphorus are required for skeletal mineralization, while other elements are involved in enzyme activity, osmotic balance, oxygen transport and antioxidant defence. Trace elements such as Fe, Cu, Zn and Mn support several metabolic and developmental processes during embryo growth (Richards, 1991; Uni et al., 2012; Torres and Korver, 2018). Changes in mineral concentration and absolute content in the yolk may therefore reflect both embryo uptake and the contribution of other egg compartments, including albumen and eggshell reserves.

The initial composition of the yolk is influenced by breeder age, genotype, egg characteristics and maternal diet. Dietary lipid source can modify the fatty acid profile of the yolk and of newly hatched chicks, while the availability of essential fatty acids during embryogenesis may affect early post-hatch metabolism (Latour et al., 1998; Cherian, 2015). In the same way, the mineral supply of breeder diets contributes to the mineral reserves deposited in the egg and may influence embryo development and chick quality (Yair and Uni, 2011; Torres and Korver, 2018). For this reason, information on breeder diet composition is useful when interpreting changes in yolk nutrient profiles during incubation. Although previous studies have provided important information on lipid mobilization, fatty acid changes and mineral transfer during turkey embryonic development, much of the turkey-specific literature was published several decades ago and generally focused on a limited number of nutrients (Couch et al., 1973; Ding et al., 1995; Ding and Lilburn, 1996; Richards, 1991, 1997; Reidy et al., 1998; Surai et al., 1999). Moreover, to our knowledge, no previous study has simultaneously evaluated both the mineral profile and fatty acid composition in the same yolk–yolk sac complex samples across different stages of turkey embryonic development. Therefore, the novelty of the present study lies in the simultaneous assessment of a broad mineral panel, including both concentrations and absolute contents, together with the relative fatty acid profile of the turkey yolk–yolk sac complex across several incubation stages, using high-precision analytical methods.

## Materials and methods

### Breeders’ management and eggs collection

A total of 200 fertile eggs were obtained from a local population of native turkeys (*Meleagris gallopavo*), aged 56 weeks, reared under conventional farm conditions in Meshgin City, Iran. The breeder flock was maintained in a semi-intensive management system with natural photoperiod, floor housing, and ad libitum access to water, and was fed a commercial turkey breeder diet commonly used in the region. Ingredients and declared chemical composition were obtained from the commercial feed label. Mineral values were obtained from label information when available and otherwise calculated from the declared formulation and standard feed composition tables, whereas the fatty acid profile of the diet was analytically determined by GC-FID. Details are reported in Table 1. Individual eggs were weighed and afterwards placed in the incubator under standard temperature and humidity conditions. Dirty, cracked or deformed eggs were removed before the incubation and the remaining eggs were weighed individually (average weight, 70.85±0.9). The room temperature was maintained at more than 25°C ten hours prior to incubation.

**Table 1:** Ingredients, declared chemical composition, mineral profile and analysed fatty acid profile of the turkey breeder diet.

Item	Value	Item	Value
Ingredients, g/kg as fed		Chemical composition	
Maize grain	475.0	AMEn, kcal/kg	2935
Wheat	90.0	AMEn, MJ/kg	12.28
Soybean meal, 44% CP	220.0	Crude protein, g/kg DM	168
Sunflower meal	45.0	Ether extract, g/kg DM	61
Soybean oil	15.0	Crude fibre, g/kg DM	38
Vegetable fat blend	20.0	Ash, g/kg DM	110
Wheat bran	28.2	Lysine, g/kg DM	8.2
Limestone	64.0	Methionine, g/kg DM	4.2
Dicalcium phosphate	17.0	Methionine + cysteine, g/kg DM	6.5
Sodium chloride	3.5	Threonine, g/kg DM	5.6
Sodium bicarbonate	1.0	Linoleic acid, g/kg diet	24.3
Vitamin–mineral premix	5.0		
DL-methionine	1.8	**Mineral profile**	
L-lysine HCl	0.8	Calcium, g/kg DM	30.0
L-threonine	0.4	Total phosphorus, g/kg DM	6.8
Choline chloride	1.0	Available phosphorus, g/kg DM	4.6
Phytase	0.1	Sodium, g/kg DM	1.9
Antioxidant	0.2	Potassium, g/kg DM	7.8
Mycotoxin binder	2.0	Magnesium, g/kg DM	2.0
Inert filler/carrier	10.0	Iron, mg/kg DM	150
**Total**	**1000.0**	Zinc, mg/kg DM	100
		Manganese, mg/kg DM	110
		Copper, mg/kg DM	15
		Selenium, mg/kg DM	0.30
**Fatty acid profile of dietary lipid fraction**	**g/kg diet**		**% total FA**
C14:0, myristic acid	0.26		0.44
C16:0, palmitic acid	13.40		22.16
C16:1, palmitoleic acid	0.10		0.17
C17:0, heptadecanoic acid	0.06		0.10
C18:0, stearic acid	2.10		3.47
C18:1 n-9, oleic acid	18.12		29.97
C18:2 n-6, linoleic acid	24.33		40.23
C18:3 n-3, α-linolenic acid	1.76		2.91
C20:0, arachidic acid	0.22		0.36
C20:1, eicosenoic acid	0.12		0.19
C20:4 n-6, arachidonic acid	Trace/not detected		—

AMEn = apparent metabolizable energy corrected for nitrogen; DM = dry matter; FA = fatty acids. Ingredients and declared chemical composition were obtained from the commercial feed label. Mineral values were obtained from label information when available and otherwise calculated from the declared formulation and standard feed composition tables. The fatty acid profile of the diet was analytically determined by GC-FID. Strontium was not supplemented and was therefore not included in the calculated mineral profile.

### Eggs incubation and sample collection

The incubation period for turkey eggs was 28 days, with a temperature of 37.7-38°C and humidity of 60-65% during the incubation stage, and a temperature of 37.5°C and humidity of 65% during hatching. Eggs were turned 90° every 6 hours, corresponding to four turning events per day, according to the routine operating protocol of the incubator, it was applied uniformly throughout the incubation period. Samples were collected at days 0, 8.5, 18.5, 23.5 and 28. The selected sampling days were chosen to represent biologically distinct stages of turkey embryonic development rather than equally spaced time intervals. These stages correspond to the pre-incubation baseline, early embryogenesis, rapid embryonic growth, late pre-hatch development, and hatch, respectively. At each time point, defective and non-embryonic eggs were identified and excluded, and 30 eggs from different positions within the incubator were selected. At days 0, 8.5, 18.5 and 23.5, the yolk with the yolk sac membrane (egg yolk–yolk sac complex, Y-YSCx) was collected after embryo removal by creating a small hole at the larger end of the egg, where the air sac is located. At day 28, samples were collected from embryos/poults at hatch before complete yolk sac absorption, and the residual Y-YSCx was carefully separated after embryo removal. The Y-YSCx collected from five eggs were pooled to obtain one experimental replicate. This pooling strategy was adopted to reduce inter-egg variability within each incubation stage and to obtain a sufficient amount of homogeneous material for both mineral and fatty acid analyses, particularly at the later stages of incubation when the residual Y-YSCx was reduced. Six pooled replicates were prepared for each incubation time. The samples were stored at −20°C until laboratory analysis.

The study involved the incubation of fertile turkey eggs and the collection of yolk–yolk sac complex samples at different stages of embryonic development. All procedures were conducted in accordance with institutional guidelines and with the Guide for the Care and Use of Agricultural Animals in Research and Teaching (FASS, 2020).

### Mineral analysis

Determination of calcium, copper, iron, magnesium, manganese, sodium, potassium, strontium, and zinc levels in Y-YSCx samples required homogenization, followed by accurate weighing, using an analytical balance with a readability of 0.0001 g, and freeze-drying. The samples were then weighed again to determine the dry Y-YSCx mass. Subsequently, they were ground to obtain a powder-like form, and 0.5 g of each sample were mixed with 5 ml of nitric acid and incubated in an oven at 50°C for 2 h and then at 90°C for 30 min. After cooling, 2 ml of hydrogen peroxide were added. The samples were heated again at 90°C for 10 min and subsequently at 120°C for 30 min. Finally, the digested samples were diluted with 30 ml of distilled water and analysed by Inductively Coupled Plasma Optical Emission Spectrometry (ICP-OES; Agilent 700 Series ICP Optical Emission Spectrometers).

### Fatty acid analysis

A total of 3 g of Y-YSCx sample was transferred into 50-mL tubes. Total lipids were extracted by adding 30 mL of chloroform:methanol solution (2:1, v/v), according to Folch et al. (1957). The tubes were vortexed for 1 min and left overnight at room temperature. The extracts were then filtered through Whatman filter paper into 100-mL volumetric flasks and rinsed twice with 8 and 4 mL of chloroform:methanol solution, respectively. Subsequently, 10 mL of 0.88% NaCl solution were added to each sample to allow phase separation. The upper phase was discarded, whereas the lower phase was transferred to glass tubes and evaporated under nitrogen or using a rotary evaporator. The dried lipid extracts were methylated with boron trifluoride in methanol at 90°C for 60 min. After cooling, 3 mL of hexane and 5 mL of distilled water were added, and the samples were vortexed and allowed to separate into two phases. The upper hexane layer, containing fatty acid methyl esters (FAME), was collected and transferred to GC vials. Fatty acid methyl esters were analysed using a GC-FID system (Agilent 7890B GC System, Agilent Technologies, Santa Clara, CA, USA) equipped with an SP-2560 capillary column (100 m × 0.25 mm i.d., 0.20 μm film thickness). Helium was used as carrier gas at a constant flow rate of 1.0 mL/min. Samples were injected in split mode at a split ratio of 1:100, with an injection volume of 1 μL. The injector and detector temperatures were set at 250°C and 260°C, respectively. The oven temperature program was as follows: initial temperature 70°C, held for 4 min; increased to 175°C at 13°C/min and held for 27 min; then increased to 215°C at 4°C/min and held for 31 min. FAME were identified by comparing their retention times with those of the Supelco 37 Component FAME Mix (Sigma-Aldrich Co., St. Louis, MO, USA). Results were expressed as percentage of total identified fatty acids; therefore, the analysis describes changes in the relative fatty acid profile and does not quantify changes in the absolute amount of individual fatty acids. The same analytical procedure was applied to a representative sample of the breeder diet to determine its fatty acid profile.

### Statistical analysis

Data were analysed using the GLM procedure of SAS software. Each pooled sample, obtained from five eggs, was considered the experimental unit. For each incubation time, six pooled replicates were analysed. Mineral concentrations, absolute mineral contents and fatty acid percentages were analysed separately using a one-way ANOVA model, with incubation day as the fixed effect. The statistical model was as follows:

$$Y_{ij}=\mu +D_{i}+e_{ij}$$

where Y_ij_ is the dependent variable, μ is the overall mean, D_i_ is the fixed effect of incubation day (0, 8.5, 18.5, 23.5 and 28 days), and e_ij_ is the residual error. Residuals were checked for normality and homogeneity of variance before analysis. When the effect of incubation day was significant, means were separated using Tukey’s test. Results are presented as means and standard error of the mean (SEM). Significance was declared at P < 0.05.

## Results

### Changes in the physical characteristics of the yolk–yolk sac complex during incubation

The wet and dry masses of the Y-YSCx changed progressively throughout incubation (Table 2). Wet Y-YSCx mass decreased markedly from the beginning of incubation to hatch, reflecting the continuous utilization of yolk reserves by the developing embryo. Dry Y-YSCx mass also declined during incubation, although at a lower rate than wet mass, while water content changed substantially across developmental stages. These changes provide important context for interpreting mineral concentrations (mg/kg) and absolute mineral contents (mg), since progressive changes in tissue mass and water content influence concentration-based measurements independently of the total amount of each mineral remaining in the Y-YSCx.

**Table 2:** Wet and dry mass of the turkey yolk–yolk sac complex during incubation.

Incubation day	Wet Y-YSCx (g)	Dry Y-YSCx (g)	Water content (g)
0	30.30 ± 1.10	14.82 ± 0.45	15.47 ± 0.80
8.5	35.91 ± 1.02	10.58 ± 0.76	24.32 ± 0.36
18.5	27.72 ± 0.80	10.23 ± 0.14	18.49 ± 0.81
23.5	22.93 ± 0.84	8.15 ± 0.67	10.08 ± 0.59
28	18.42 ± 2.31	8.97 ± 2.05	8.15 ± 0.39

### Changes in mineral profile during embryo development

The mineral concentrations of the turkey egg Y-YSCx at different incubation times are reported in Table 3. Incubation time significantly affected the concentrations of Ca, Cu, Fe, K, Na, P, Sr and Zn (P < 0.0001), whereas Mg and Mn concentrations were not affected (P = 0.4371 and P = 0.9367, respectively). Calcium concentration increased progressively during incubation, with the lowest values observed at days 0 and 8.5 and the highest value recorded at day 28. Strontium showed a similar increasing pattern, rising from 2.74 mg/kg at day 0 to 8.54 mg/kg at day 28. In contrast, phosphorus concentration decreased markedly as incubation progressed, from 7246.6 mg/kg at day 0 to 2945.2 mg/kg at day 28. Copper concentration increased transiently at day 8.5, reaching the highest value, and then returned to values comparable with those observed at day 0. A similar early increase was observed for Fe, K, Na and Zn, which reached their highest concentrations at day 8.5 and subsequently decreased during the later stages of incubation, showing their lowest concentrations at day 28, The absolute mineral contents of the turkey egg Y-YSCx are presented in Table 4. Incubation time significantly affected the absolute contents of Ca, Cu, Fe, K, Mg, Na, P, Sr and Zn, whereas Mn was not significantly affected (P = 0.0549). Calcium showed a distinct pattern compared with most other minerals. Its absolute content decreased from day 0 to day 8.5, but then increased progressively throughout incubation, reaching the highest value at day 28. In contrast, phosphorus showed a continuous decrease from day 0 to hatching, declining from 107.15 mg to 24.66 mg. Iron also decreased markedly during incubation, with the highest value observed at day 0 and the lowest value at day 28. Copper, potassium, sodium and zinc showed a transient increase during the early phase of incubation. Their absolute contents reached the highest values at day 8.5 and subsequently decreased during the later stages. At day 28, Cu, K, Na and Zn contents were lower than the values observed at the beginning of incubation. Magnesium showed moderate changes during incubation, with lower values observed at days 18.5 and 28 compared with day 0. Strontium content increased transiently and reached its highest value at day 23.5, before returning at day 28 to values comparable with those observed at the other incubation times.

**Table 3:** Mineral concentrations of the turkey egg yolk–yolk sac complex at different incubation times.

Mineral(mg/kg)	Embryonic day					SEM	P-value
	0	8.5	18.5	23.5	28		
*Ca*	2437.1^d^	2541.3^d^	4405.3^c^	7976.3^b^	8947.3^a^	171.2	>0.0001
*Cu*	7.80^b^	16.62^a^	7.67^b^	9.08^b^	8.10^b^	0.82	>0.0001
*Fe*	308.22^b^	333.38^a^	98.20^cd^	110.95^c^	85.36^d^	7.98	>0.0001
*K*	1704.1^b^	2388.5^a^	1860.1^b^	1718.5^b^	548.3^c^	61.8	>0.0001
*Mg*	175.33	177.11	175.15	212.51	208.32	4.89	=0.4371
*Mn*	1.71	1.87	1.90	1.63	1.87	0.36	=0.9367
*Na*	1038.6^bc^	3120.5^a^	1151.4^b^	925.8^bc^	805.0^c^	72.6	>0.0001
*P*	7246.6^a^	6414.4^ab^	5720.7^b^	3468.0^c^	2945.2^c^	1142.9	>0.0001
*Sr*	2.74^d^	3.92^c^	6.07^b^	7.57^b^	8.54^a^	0.67	>0.0001
*Zn*	53.58^b^	78.54^a^	56.08^b^	54.44^b^	32.93^c^	4.99	>0.0001

Within each row, means with different superscript letters differ significantly (P < 0.01), where the overall effect of incubation day was significant.

SEM = standard error of the mean.

**Table 4:** Absolute mineral contents of the turkey egg yolk–yolk sac complex at different incubation times.

Mineral	Embryonic day					SEM	P-value
	0	8.5	18.5	23.5	28		
Ca (mg)	36.04^d^	29.05^e^	54.03^c^	61.03^b^	82.04^a^	5.69	>0.0001
Cu (µg)	115.48^b^	189.97^a^	78.47^c^	108.28^b^	67.63^c^	8.96	>0.0001
Fe (mg)	4.56^a^	3.81^b^	1.01^d^	1.32^c^	0.71^e^	0.04	>0.0001
K (mg)	27.18^b^	60.11^a^	26.18^b^	16.45^c^	4.35^d^	0.36	>0.0001
Mg (mg)	2.59^a^	2.02^ab^	1.79^b^	2.53^ab^	1.75^b^	0.21	=0.0308
Mn (µg)	25.27	21.42	18.41	19.46	15.64	3.98	=0.0549
Na (mg)	15.36^b^	35.67^a^	11.77^c^	11.03^c^	7.74^d^	2.98	>0.0001
P (mg)	107.15^a^	73.31^b^	58.47^c^	41.32^d^	24.66^e^	8.24	>0.0001
Sr (µg)	72.41^b^	76.24^b^	78.14^b^	101.82^a^	71.90^b^	6.57	>0.0001
Zn (mg)	0.79^ab^	0.89^a^	0.57^c^	0.65^bc^	0.27^d^	0.04	>0.0001

Within each row, means with different superscript letters differ significantly (P < 0.05).

SEM = standard error of the mean.

### Changes in fatty acid profile during embryo development

The relative fatty acid composition of the turkey egg Y-YSCx at different incubation times is reported in Table 5. Incubation time significantly affected the proportions of all the fatty acids considered (P < 0.01). Palmitic and oleic acids were the predominant fatty acids throughout incubation, followed by stearic, palmitoleic and arachidonic acids. Among saturated fatty acids, palmitic acid showed a moderate increase during incubation and reached its highest relative proportion at day 28. Myristic acid remained relatively stable from day 0 to day 23.5, but increased markedly at hatching. Heptadecanoic acid increased during the first half of incubation, reaching the highest relative value at day 18.5, and then declined to values comparable with those observed at day 0. Stearic acid fluctuated during incubation, with the highest relative value observed at day 23.5 and the lowest relative values recorded at days 8.5 and 28. Among monounsaturated fatty acids, palmitoleic acid increased from day 0 to days 8.5 and 18.5, decreased at day 23.5, and returned at day 28 to values comparable with those observed at day 0. Oleic acid also showed marked changes, decreasing at day 8.5, increasing progressively up to day 23.5, and then reaching the lowest relative value at day 28. Regarding polyunsaturated fatty acids, linoleic acid increased overall during incubation, with the highest proportion recorded at day 28. α-Linolenic acid showed only a transient decrease at day 8.5, whereas values at the other incubation times were comparable. In contrast, arachidonic acid decreased markedly during incubation, from 4.80% at day 0 to 0.17% at day 28.

**Table 5:** Fatty acid composition of the turkey egg yolk–yolk sac complex at different incubation times.

Fatty Acids (%)	Embryonic day					SEM	P-value
	0	8.5	18.5	23.5	28		
Myristic acid	1.61^b^	1.40^b^	1.16^b^	0.84^b^	3.75^a^	0.84	=0.0014
Palmitic acid	36.44^c^	37.47^bc^	39.08^b^	38.00^bc^	49.83^a^	3.25	<0.0001
Palmitoleic acid	6.25^b^	8.66^a^	9.02^a^	4.66^c^	6.76^b^	0.45	<0.0001
Heptadecanoic acid	0.14^c^	1.22^ab^	1.35^a^	1.14^b^	0.23^c^	0.01	<0.0001
Stearic acid	9.12^ab^	6.41^c^	8.27^b^	10.03^a^	5.88^c^	0.48	=0.0003
Oleic acid	34.68^c^	31.02^d^	36.04^b^	39.08^a^	25.93^e^	1.84	<0.0001
Linoleic acid	1.96^d^	3.58^b^	2.36^c^	3.40^b^	4.42^a^	0.42	=0.0028
Linolenic acid	1.03^a^	0.29^b^	1.30^a^	1.09^a^	1.18^a^	0.14	=0.0034
Arachidonic acid	4.80^a^	1.26^b^	1.84^b^	1.50^b^	0.17^c^	0.02	<0.0001

Within each row, means with different superscript letters differ significantly (P < 0.05). SEM = standard error of the mean. Values are expressed as percentage of total identified fatty acids.

## Discussion

### Changes in mineral profile during embryo development

The egg yolk is considered the main storage site for most minerals and represents a major mineral source for the developing embryo during incubation (Richards and Packard, 1996). Therefore, measure the changes in mineral concentrations and absolute contents in the Y-YSCx provide useful information on the timing and extent of mineral mobilization during embryo development. Moreover, the progressive reduction in wet and dry Y-YSCx mass observed during incubation further supports the interpretation of the mineral data. Because mineral concentrations are expressed relative to tissue mass, reductions in Y-YSCx mass and changes in water content may increase the measured concentration of some minerals even when their absolute amounts remain stable or decrease. For this reason, considering both concentrations and absolute contents helps distinguish changes in mineral density from changes in the total quantity of each mineral remaining in the Y-YSCx.

In the present study, most minerals showed a decline in absolute content by hatch, whereas Ca and Sr followed different patterns. These results are consistent with the high mineral demand of the embryo during mid and late incubation, when tissue growth, skeletal mineralization and preparation for hatching become more intense (Yadgary et al., 2010).

Calcium showed a distinct trend compared with the other minerals. Its concentration and absolute content were highest at hatch. However, during the early phase of incubation, Ca concentration remained relatively stable, whereas its absolute content decreased at day 8.5. This difference partly reflects the reduction in dry Y-YSCx mass and is also consistent with the transfer of yolk Ca during early embryonic development. This is consistent with the observation that during the first 7 to 10 days of incubation, the yolk contributes to the calcium supply of the embryo before the eggshell becomes the main source (Richards and Packard, 1996). The subsequent increase in Ca content from the middle to the final stage of incubation agrees with previous studies on turkey, chicken and goose eggs (Richards, 1991; Hopcroft et al., 2019; Besharati et al., 2023). This increase is likely related to the mobilization of calcium from the eggshell through the chorioallantoic membrane, which becomes fully functional during the second week of incubation (Johnston and Comar, 1955; Crooks and Simkiss, 1974; Gabrielli et al., 2004; Gabrielli and Accili, 2010). Strontium also showed an increase in concentration throughout incubation, while its absolute content increased only transiently, reaching the highest value at day 23.5 and then returning at hatch to values comparable with those observed at the other incubation times. This pattern may indicate a partial transfer of Sr from the eggshell to the Y-YSCx during the second half of incubation. A similar increase in yolk Sr concentration was reported in chicken embryos by Hopcroft et al. (2019), although their observations did not extend to hatch. In goose eggs, Besharati et al. (2023) observed an increase in Sr concentration but a reduction in absolute content during the second half of incubation. Taken together, these findings may suggest that Sr may follow a pattern partly associated with eggshell mineral mobilization, although its physiological role in avian embryo development remains poorly defined. Copper, Fe and Zn showed dynamic changes during incubation. Their concentrations increased at day 8.5 and then decreased during the later stages. However, changes in concentration were not always reflected in the corresponding absolute contents, indicating that concentration data alone may not fully describe mineral dynamics in the residual Y-YSCx. For Fe, the higher concentration observed at day 8.5 was not accompanied by an increase in absolute content, which instead declined. This discrepancy is consistent with the lower dry Y-YSCx mass at this stage and highlights the importance of interpreting concentration and absolute content jointly. In contrast, Cu showed a true transient accumulation in absolute content at day 8.5, reaching its highest value. A similar early increase in yolk Cu content was reported by Besharati et al. (2023) in goose eggs. This pattern may reflect redistribution of Cu among egg compartments during early incubation, when albumen components are progressively incorporated into the amniotic fluid and become available to the embryo (Romanoff, 1967). Zn exhibited an intermediate pattern, with increased concentration at day 8.5 but only a modest change in absolute content, indicating a partial decoupling between concentration and total amount. The marked reduction in Fe, Cu and Zn contents during the second half of incubation is consistent with the increasing demand for these trace elements during embryonic development. Similar trends have been described in turkey (Richards, 1991), chicken (Yair and Uni, 2011; Hopcroft et al., 2019), duck (Onbaşılar et al., 2014) and goose (Besharati et al., 2023). These elements have well-established biological roles. Zinc is involved in immune development, hepatocyte maturation and bone formation (Benzo and de la Haba, 1972; Kidd et al., 1992; Oviedo-Rondon and Ferket, 2005; Dibner et al., 2007). Copper is required for iron metabolism, haemoglobin synthesis, cytochrome oxidase activity and connective tissue development (Dibner et al., 2007; Leeson, 2009). Iron is essential for oxygen transport and cellular energy metabolism, mainly as a component of haemoglobin, myoglobin and several redox enzymes (Richards, 1997). Therefore, the reduction in their residual Y-YSCx content at hatch is biologically consistent with the increasing requirement for oxygen transport, tissue growth and metabolic activity during late embryogenesis. Sodium and K also showed a clear transient increase during early incubation, followed by a marked decline toward hatch, in agreement with previous observations in chicken and goose embryos (Hopcroft et al., 2019; Besharati et al., 2023). Because albumen represents an important source of these ions, the early increase may reflect redistribution among egg compartments (Richards and Packard, 1996; Richards, 1997; Uni et al., 2012), whereas the subsequent decline is consistent with their progressive use during embryo development. Magnesium and Mn were the only elements that did not show significant changes in concentration during incubation. The absolute content of Mg changed moderately over time, whereas Mn showed only a tendency to decrease. These results suggest a more gradual or limited mobilization of these elements compared with P, Fe, Cu and Zn. Similar patterns for Mn have been reported in chicken and goose eggs (Hopcroft et al., 2019; Besharati et al., 2023). In turkey, Richards (1991) observed a decrease in yolk Mn concentration between days 22 and 26 of incubation, together with an increase in the yolk sac, suggesting active mobilization from yolk reserves. Mg is involved in skeletal development and antioxidant protection during embryogenesis (Leach et al., 1969; Liu et al., 1994; Qin et al., 2017; Geng et al., 2022). Mg may also contribute to antioxidant defence, as Mg deficiency has been shown to increase hydrogen peroxide production and reduce catalase activity in chick embryo hepatocytes (Yang et al., 2005). The relatively stable concentrations of Mg and Mn, despite changes in dry Y-YSCx mass, were accompanied by moderate changes in their absolute contents. This further illustrates that stable concentrations do not necessarily indicate stable amounts within the residual Y-YSCx. Phosphorus showed one of the clearest trends, with both concentration and absolute content decreasing markedly throughout incubation. This indicates substantial mobilization of yolk P by the developing embryo. A similar decline has been reported in goose (Besharati et al., 2023), chicken (Yair and Uni, 2011; Hopcroft et al., 2019) and duck eggs (Onbaşılar et al., 2014). The yolk is the main source of P for the embryo, and this reserve is progressively mobilized through the yolk sac membrane as incubation proceeds (Yair and Uni, 2011). The strong reduction observed in the present study is consistent with the central role of P in skeletal development and energy metabolism during late embryogenesis (Li et al., 2014).

Overall, the mineral results indicate that the turkey Y-YSCx is a dynamic matrix whose composition changes according to the developmental stage of the embryo. With the exception of Ca and, to a lesser extent, Sr, most mineral reserves in the residual Y-YSCx were reduced by hatch. This suggests that the late embryonic period is characterized by intense mineral mobilization, which may contribute to supporting rapid growth, skeletal mineralization, metabolic activity and preparation for hatching.

### Changes in fatty acid profile during embryo development

Avian yolk fatty acids occur mainly in esterified form within triacylglycerol and phospholipid fractions. During incubation, yolk lipids are absorbed by the yolk sac membrane, processed within the yolk sac tissue and transferred to the embryo through the circulation. Triacylglycerols are mainly used as an energy source, whereas phospholipids provide structural components for membrane formation (Speake et al., 1998). In turkey embryos, approximately 80% of yolk lipids are mobilized during embryogenesis, while the remaining fraction forms the residual yolk that is internalized at hatch and supports the chick during the early post-hatch period (Ding et al., 1995; Ding and Lilburn, 1996). The greatest proportion of yolk lipid transfer in turkey occurs during the final days of incubation, when embryo growth and energy demand are highest (Ding et al., 1995). In the present study, the fatty acid profile of the Y-YSCx changed substantially during incubation. Since fatty acids were expressed as percentages of total identified fatty acids, these results should be interpreted as changes in relative proportions rather than direct measurements of absolute fatty acid utilization. Nevertheless, the observed shifts provide useful information on the remodelling of the residual yolk lipid fraction during turkey embryogenesis. The increase in the relative proportion of myristic and palmitic acids at hatch, together with the decrease in oleic and arachidonic acids, suggests that individual fatty acids do not remain in the residual Y-YSCx in the same proportions throughout development. These changes may reflect the combined effects of embryo uptake, yolk sac lipid processing, hydrolysis and re-esterification reactions, and the changing composition of the residual lipid pool. The behaviour of palmitic acid was particularly clear. It increased during incubation and reached the highest relative proportion at hatch. This relative increase does not necessarily mean that palmitic acid was not used by the embryo. Rather, it shows that palmitic acid represented a greater proportion of the residual fatty acid pool at hatch, which may have resulted from differential changes in the relative abundance of the other fatty acids. Ding and Lilburn (1996) reported a relatively conservative pattern of palmitic acid use during turkey embryogenesis, followed by a marked reduction after hatch. This supports the hypothesis that part of the palmitic acid remaining in the residual yolk may still be available for early post-hatch metabolism. A similar relative pattern was observed for myristic acid, whose proportion remained relatively stable until day 23.5 and increased sharply at hatch. Oleic acid was the predominant monounsaturated fatty acid and showed a different pattern. Its relative proportion increased up to day 23.5 and then decreased markedly at hatch. This trend is partly consistent with previous work in turkey and chicken embryos, where oleic acid was one of the main fatty acids involved in yolk lipid transfer and tissue deposition (Couch et al., 1973; Ding and Lilburn, 1996; Reidy et al., 1998; Speake et al., 1998; Sahan et al., 2014). The reduction observed in the relative proportion of oleic acid at hatch may indicate a greater relative removal or transformation of oleic acid during the final stage of incubation, when lipid oxidation and tissue accretion are particularly active. However, it should also be noted that the yolk sac membrane is not a passive transfer surface. It can participate in fatty acid esterification and lipid restructuring before transfer to the embryo (Powell et al., 2004). Therefore, the changes in oleic acid may reflect not only embryo uptake, but also lipid processing within the yolk sac membrane. Several studies have shown that yolk lipid transport involves hydrolysis and re-esterification processes during passage through the yolk sac membrane (Speake et al., 1998; Powell et al., 2004). Speake et al. (1998) also reported an enrichment of oleic acid in cholesteryl esters within the yolk sac membrane and embryonic liver during mid and late development. This may explain why oleic acid often increases during embryogenesis in different avian species. In the present study, however, the final decrease at hatch could be suggest that the balance between oleic acid retention, transformation and transfer changed during the last phase of incubation. Differences in breeder age (Latour et al., 1998), egg composition and dietary lipid supply (Cherian, 2015) may have contributed to this pattern. Among polyunsaturated fatty acids, arachidonic acid showed a marked decline from day 0 to hatch. This pattern suggests that arachidonic acid became proportionally less abundant in the residual Y-YSCx as incubation progressed. Arachidonic acid has an important role in membrane phospholipids and in the development of tissues with high metabolic activity, including heart, liver, kidney and brain (Speake et al., 1998; Decrock et al., 2002). The marked reduction in the relative proportion of arachidonic acid is consistent with its involvement in developing tissues. However, absolute fatty acid data would be required to determine whether this pattern resulted from preferential transfer, utilization, or changes in the proportions of the other fatty acids. The low relative proportion of arachidonic acid observed at hatch is compatible with previous evidence described in avian embryos, according to which long-chain PUFA are preferential incorporated into developing embryonic tissues (Surai et al., 1999; Speake et al., 1998). Linoleic acid showed an overall increase in relative proportion, reaching its highest value at hatch, whereas α-linolenic acid remained relatively stable, except for a transient decrease at day 8.5. This suggests that these precursor fatty acids may have been relatively conserved in the residual Y-YSCx compared with arachidonic acid. Linoleic acid is the precursor of arachidonic acid, while α-linolenic acid is the precursor of longer-chain n-3 PUFA, including docosahexaenoic acid. Their persistence in the residual yolk may be relevant for early post-hatch metabolism, when the chick continues to rely partly on residual yolk nutrients. However, because only relative percentages were measured in the present study, this interpretation should be considered cautiously. Absolute fatty acid contents would be needed to determine whether these fatty acids were truly conserved or whether their higher relative proportions resulted from the greater reduction of other fatty acids.

The fatty acid profile of the breeder diet was analytically determined to provide a descriptive background for interpreting yolk fatty acid composition. However, because only one dietary treatment was considered, no causal relationship can be established between maternal lipid supply and the changes observed during incubation. Future studies should include total lipid content and absolute fatty acid measurements to better characterize fatty acid changes in the Y-YSCx.

Overall, these results show that the fatty acid profile of the turkey Y-YSCx is progressively remodelled during incubation. The observed changes suggest that the proportions of individual fatty acids in the residual Y-YSCx varies according to embryonic stage, late developmental requirements and possible early post-hatch needs. Although the absence of absolute fatty acid contents limits the interpretation of selective utilization, the present data provide useful descriptive information on the remodelling of the residual yolk fatty acid profile during turkey embryonic development and contribute to enrich the limited literature available for this species.

## Conclusion

The present study provides a comprehensive description of changes in mineral and fatty acid composition of the turkey egg Y-YSCx during embryonic development. The results showed that incubation was associated with marked changes in mineral dynamics, with Ca and Sr showing an increase toward the final stages of development, whereas P, Fe, Cu, K, Na and Zn generally decreased in absolute content by hatch. These patterns confirm the active role of the Y-YSCx as a dynamic extraembryonic compartment involved in nutrient storage, mobilization and transfer to the developing embryo. The relative fatty acid profile also changed during incubation, with relative higher proportions of the total identified fatty acids of myristic, palmitic and linoleic acids at hatch and a marked reduction in oleic and arachidonic acids. Because fatty acids were expressed as percentages of total identified fatty acids, these changes should be interpreted as evidence of lipid remodelling rather than as direct evidence of the absolute utilization, retention, or depletion of individual fatty acids. Overall, the findings provide useful information on changes in mineral content and relative fatty acid composition during turkey embryogenesis and may support future studies aimed at clarifying the metabolic mechanisms regulating yolk nutrient mobilization, embryo development and early post-hatch chick quality.

## Ethics statement

The study involved the incubation of fertile turkey eggs and the collection of yolk–yolk sac complex samples at different stages of embryonic development. All procedures were conducted in accordance with institutional guidelines and with the Guide for the Care and Use of Agricultural Animals in Research and Teaching (FASS, 2020).

## Funding

This research received no external funding.

## Competing interest

The authors declare no competing interests.

## Authorship contribution statement

Leila Fathi: Conceptualization, Methodology, Investigation, Formal Analysis, Writing - original draft preparation. Maghsoud Besharati: Conceptualization, Methodology, Data curation, Writing - original draft praparation, Writing - review and editing. Zabihollah Nemati: Conceptualization, Methodology, Investigation, Data curation. Reza Asadpour: Investigation, Formal analysis, Writing - review and editing. Lucrezia Forte: Data curation. Aristide Maggiolino: Conceptualization, Formal analysis, Data curation, Writing - review and editing. Gerardo Centoducati: Writing - review and editing. Giusy Rusco: Conceptualization, Writing - original draft praparation, Writing - review and editing. All authors have read and agreed to the published version of the manuscript.

## Declaration of generative AI and AI-assisted technologies in the manuscript preparation process

During the preparation of this work, the authors used ChatGPT to improve readability and language. The authors reviewed and edited the output as needed and take full responsibility for the content of the published article.

## Financial Interest

None of the authors have received any financial support or have any financial interests that could have influenced the outcomes or interpretations of this research.

## Non-Financial Interests

There are no non-financial interests (such as personal or professional relationships, affiliations, or beliefs) that could be perceived to affect the objectivity or impartiality of the research reported in the manuscript.

We confirm that this manuscript has not been submitted to or is under consideration by another journal. All authors have approved the manuscript and agree with its submission to Poultry Science Journal.

We declare that the data presented in this manuscript is original, accurate, and has not been fabricated or manipulated. Any conflicts of interest, financial or otherwise, have been disclosed and acknowledged.

## Disclosures

We, the undersigned authors of the manuscript, declare that we have no conflicts of interest related to the research, authorship, and/or publication of this article.
